# Infrared ship target detection algorithm PEW_YOLOv8 in complex environments

**DOI:** 10.1038/s41598-026-40574-8

**Published:** 2026-02-23

**Authors:** Tingkai Dong, Menglin Zhu, Gaofeng Tang

**Affiliations:** 1 School of Software, Henan University of Engineering, Zhengzhou, 451191 Henan China; 2 Institute of Big Data And Artificial Intelligence, Zhengzhou University of Science and Technology, Zhengzhou, 450064 Henan China; 3 School of Computer Science and Information Security, Guilin University of Electronic Technology, Guilin, 541004 Guangxi China

**Keywords:** Deep learning, Target recognition, Infrared images, YOLOv8, Engineering, Mathematics and computing

## Abstract

In the infrared ship detection task under complex environments, issues such as high rates of missed and false detections occur due to noise, occlusion, and the indistinct features of small targets. To address these problems, this paper proposes a ship target detection algorithm, PEW_YOLOv8, based on YOLOv8. Firstly, the FFA-Net algorithm is used for image pre-processing to improve the contrast and clarity of the images. Secondly, the PGIG-Backbone network is designed. Through multi-path fusion technology, it ensures that features at different scales can complement each other, enhancing the detail expression ability of small targets. Subsequently, the enhanced multi-scale attention neck network, EMA-Neck, is designed. By means of the attention mechanism, it suppresses background noise, enhances feature information related to the target, and improves the distinguishability between the target and the background. Finally, the WIoU Loss is introduced. Through a more comprehensive method of evaluating bounding boxes, the model can better handle inter-target overlaps, occlusions, and other interferences in complex scenes. Under the same experimental conditions, compared with YOLOv8, the PEW_YOLOv8 algorithm achieves a detection accuracy of 92.2% on the Raytron Technology infrared ship dataset, increasing mAP50 and mAP50:95 by 3.9% and 3.1% respectively.

## Introduction

Maritime ship target detection can enhance the safety and efficiency of maritime traffic. Real-time monitoring technology based on ship-borne cameras involves equipping ships navigating on water with optoelectronic platforms. Computer vision algorithms are then used to detect ship targets in the video frames returned by the cameras. Once a suspicious target is detected, an early warning is immediately issued, enabling the judgment or prediction of maritime targets and their possible behavior outcomes. This is of great strategic significance for modern maritime monitoring and management.

Infrared ship target detection has become a key technical approach for target perception in complex marine environments due to its all-weather operation capability. Its development can be divided into two stages: traditional hand-crafted feature methods and deep-learning-based methods. In the early days, traditional methods relied on hand-designed feature descriptors to characterize targets and backgrounds, such as Haar-like features, Histogram of Oriented Gradients (HOG) features, Local Binary Pattern (LBP) features, etc. Then, classifiers such as Support Vector Machine (SVM)^1^ and Adaboost^2^ were used for target recognition and detection. Alves^3^ et al. used moment invariants and neural networks to identify ship types from infrared images. Given input images from infrared or range sensors, interesting objects were found in these images and then classified. Paul Withagen et al.^4^ adopted a segmentation algorithm based on wave gray-value distribution and Hough transform to locate the waterline of ships, relating the ship size in image coordinates and the angle between the waterline and the horizon to the actual size and azimuth of the ship. With the application of deep-learning technology, infrared ship target detection can utilize models such as convolutional neural networks to automatically extract features from infrared images, accelerating the process of infrared ship recognition and detection and improving detection efficiency. In 2014, Ross Girshick, Jeff Donahueet al.^5^ proposed R-CNN, which inaugurated a new era of object detection using deep learning and had a profound impact on subsequent object-detection algorithms. Algorithms in this period can be mainly classified into two categories. One is the two-stage detection algorithms based on region proposals, such as R-CNN, Fast R-CNN^6^, and Faster R-CNN^7^. These algorithms first generate region proposals from the image and then classify these regions. The other is the one-stage detection algorithms based on regression, such as YOLO (You Only Look Once)^8^ and SSD (Single Shot MultiBox Detector)^9^. These algorithms directly predict the category and location of objects on the image.

In recent years, researchers have continuously optimized algorithms to meet the requirements of complex scenarios in infrared ship detection. Wenying et al.^10^ proposed a Two-Channel Image Separation (TCS) method. This method separates the local image of the target area into a bright-channel image and a dark-channel image, making the grayscale distribution of the target in the sub-images relatively uniform. Finally, edge-guided binarization is used to extract the target. The experimental results show that the algorithm proposed in this paper can effectively detect small and weak targets in nighttime infrared sea surface images, and its detection accuracy is superior to that of the comparative algorithms. Özertem Kemal Arda^11^ first performs morphological gray-scale reconstruction on the input image, and then conducts automatic threshold segmentation on the suppressed image. In the segmentation step, connected-component analysis is used to obtain the target candidate regions, thus obtaining the final detection results.

Xiaowo Xu^12^, aiming at the problems of variable scales and clutter interference in synthetic aperture radar (SAR) remote-sensing ship detection, proposed a comprehensive score voting method based on distribution-based anchor assignment and distance-penalty IoU loss. By optimizing the anchor-matching logic and the localization loss function, the accuracy and robustness of target detection in complex backgrounds were significantly improved, providing a direct reference for the design of anchor strategies and the optimization of loss functions in infrared ship detection. Xiaotong Li^13^ innovatively adopted a reinforcement-learning-driven joint detection and tracking framework. Through a dynamic decision-making mechanism, it can cope with the uncertainty of targets in complex environments. The idea of enhancing anti-interference ability in continuous target perception provides a new perspective for dealing with problems such as target occlusion and dynamic interference in infrared ship detection.

In the field of underwater image enhancement, Hao Wang^14^ proposed the WaterCycleDiffusion model. This model effectively addresses issues such as image blurring and color distortion through visual-text feature fusion technology. Its cross-modal feature enhancement approach offers a new perspective for feature extraction in infrared ship detection under low signal-to-noise ratios and environmental interference. Hao Wang^15^ conducted research using underwater color differences as clues and achieved color consistency optimization by exploring distortion patterns, verifying the effectiveness of "feature-difference modeling" in complex environments. This can provide a reference for correcting illumination interference in infrared ship detection. Xiaotong Li^16^, for the scene classification task of High-Frequency Surface Wave radar (HFSWR), the S2G-GCN network combines spectral graph modeling with a graph convolutional network to enhance the capture of associated information between features. This provides technical support for distinguishing features between targets and backgrounds in infrared ship detection.

Although deep-learning-based infrared ship target detection has achieved good detection accuracy, it still faces some challenges:

1. Due to overheating of infrared equipment, insufficient waterproofing, and humid air, the heat generated during the operation of infrared lamps and CCD boards can cause water mist, resulting in foggy, blurred, or distorted ship images.
2. Infrared images have low resolution and contain targets of different scales. Low resolution leads to the loss of target detail information, making it difficult for algorithms to accurately identify targets. Targets of different scales require receptive fields of different sizes to extract features. Receptive fields of the same scale may cause the features of small targets in low-resolution images to be ignored.
3. During the fusion of the neck network, due to low contrast and the diversity of target sizes, there is insufficient feature fusion and feature transfer ability.
4. Due to environmental factors such as smoke or lighting conditions, the boundaries of ships become blurred or partially occluded. However, the CIoU loss function aims to predict an accurate bounding box and performs poorly when dealing with targets with unclear boundaries.

To address these challenges, this paper proposes the PEW_YOLOv8 algorithm, and the main work is as follows:

1. To address issues such as foggy, blurred, or distorted ship images, the FFA-Net (Feature Fusion Attention Network) module is introduced, which combines channel and pixel attention with local residual learning to restore image detail information.
2. To address the information bottleneck problem caused by different scenarios and target sizes, the PGIG-Backbone network (PGI-Gradient-optimized Backbone) is proposed based on efficient spatial encoding technology, enabling the model to obtain reliable gradient information to update network weights and improve detection accuracy.
3. To address the insufficient feature fusion and extraction ability of the neck network, the efficient multi-scale attention mechanism (EMA attention) is introduced. It assigns different weights to each position in different feature maps, emphasizes features that are more important for the current task, improves the detection of small and weak targets, and enhances the model’s multi-scale object detection ability and model inference speed.
4. To address the poor performance of the base model when dealing with targets with unclear boundaries, the Wise-IoU bounding box loss function with a dynamic non-monotonic focusing mechanism is introduced. This loss function uses “outlier degree” to evaluate the quality of anchor boxes instead of simply relying on IoU values. While reducing the competitiveness of high-quality anchor boxes, it also reduces the harmful gradients generated by low-quality examples.

## Methods

### YOLOv8 algorithm

YOLOv8 (You Only Look Once v8) is an object-detection algorithm developed by Ultralytics. It is an improvement based on the YOLO series. The backbone network is responsible for extracting features from the input image. The Feature Pyramid, a multi-scale feature representation, is used to detect targets of different sizes. YOLOv8 constructs the feature pyramid by adding connections between different layers of the backbone network. The Detection Head is responsible for mapping the feature maps to bounding boxes and class probabilities. YOLOv8 uses a new Anchor-Free detection head, no longer relying on predefined anchor boxes, and finally determines the object positions and classes in the image.

### Image defogging pre-processing

**Fig. 1:**
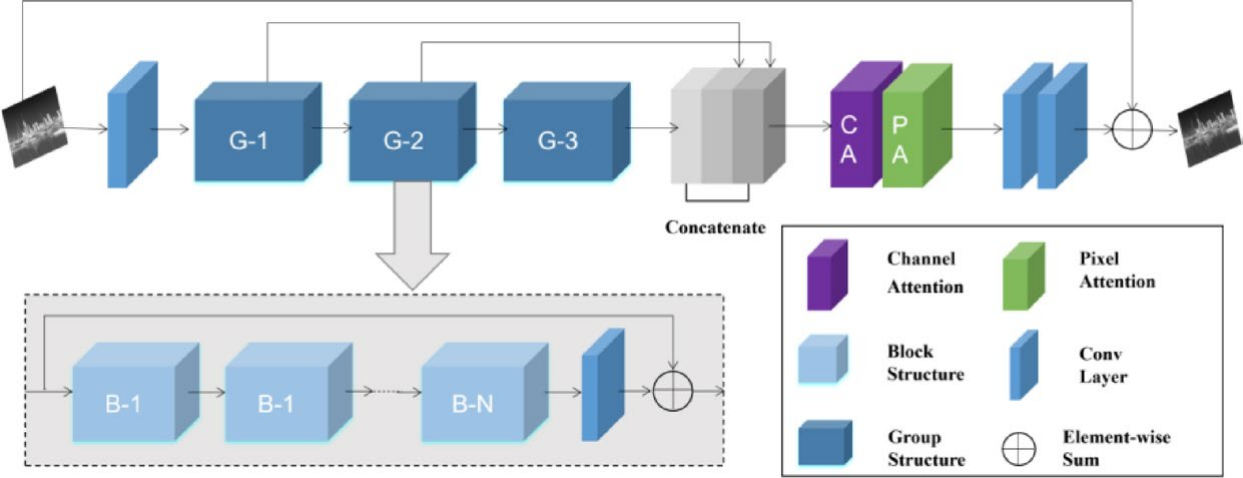
Structure of the feature fusion attention network (FFA).

Due to issues such as fogging and blurring in infrared images, the YOLOv8 algorithm fails to extract useful feature information, resulting in a decline in detection accuracy. Therefore, the FFA-Net^17^ module is introduced. FFA (As shown in Fig. 1) consists of N Group Architectures, local residual learning, and a Feature Attention module (FA).

The feature attention module is an attention-mechanism structure composed of Channel Attention (CA)^18^ and Pixel Attention (PA). The features input to the CA pass through two convolutional layers and two activation functions, sigmoid and ReLU. The calculation process of channel attention is shown in formula (1).

1 $$CA_{c} = \sigma (Conv(\delta (Conv(g_{c} ))))$$

Among them, $\sigma$ is the sigmoid function,$\delta$ is the ReLU function, and $g_{c}$ is the channel descriptor. Finally, we multiply the input Fc and the weight of the channel CAc element-by-element to obtain the output $F^{*}$ of the channel attention CA.

Pixel Attention (PA) is similar to Channel Attention (CA). The input $F^{*}$ (the output of CA) is directly fed into two convolutional layers with ReLU and sigmoid activation functions. The shape changes from *C* × *H* × *W* to *1* × *H* × *W*. The calculation process of pixel attention is shown in formula (2).

2 $$PA_{c} = \sigma (Conv(\delta (Conv(F^{*} ))))$$

Finally, an element-by-element multiplication of the input $F^{*}$ and the result of *PA* is carried out, yielding the output $\tilde{F}$ of the FA module. The calculation process of $\tilde{F}$ is presented as in Eq. (3).

3 $$\tilde{F} = F^{*} \otimes PA$$

### Programmable gradient information structure

**Fig. 2:**
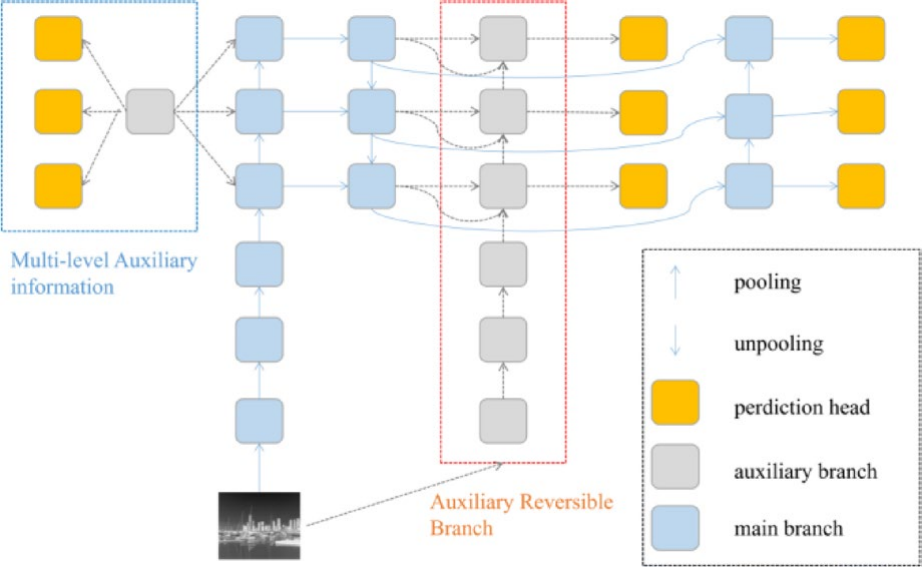
Programmable gradient information structure.

The PGI (Programable Gradient Information) structure^19^ mainly consists of three parts: the main branch, the auxiliary reversible branch, and multi-level auxiliary information, as shown in Fig. 2. The following will introduce them respectively:

1. Main branch: The main branch is composed of partial convolutions, which perform convolutional feature extraction on the input images.
2. Auxiliary reversible branch: The auxiliary reversible branch includes the CBfuse module, C2f. convolution, and ordinary convolution. The CBfuse module concatenates feature maps from different levels to form multi-scale feature maps. These feature maps are then fused through convolution operations and activation functions to obtain a more abundant feature representation.
3. Multi-level auxiliary information: It controls the main branch to learn programmable multi-level semantic information. The multi-level auxiliary information is to insert an integration network between the feature pyramid hierarchical layer of the auxiliary supervision and the main branch. Then, it is used to combine the returned gradients from different prediction heads, aggregate the gradient information containing the target objects, and transfer it to the main branch.

### Efficient multi-scale attention mechanism

**Fig. 3:**
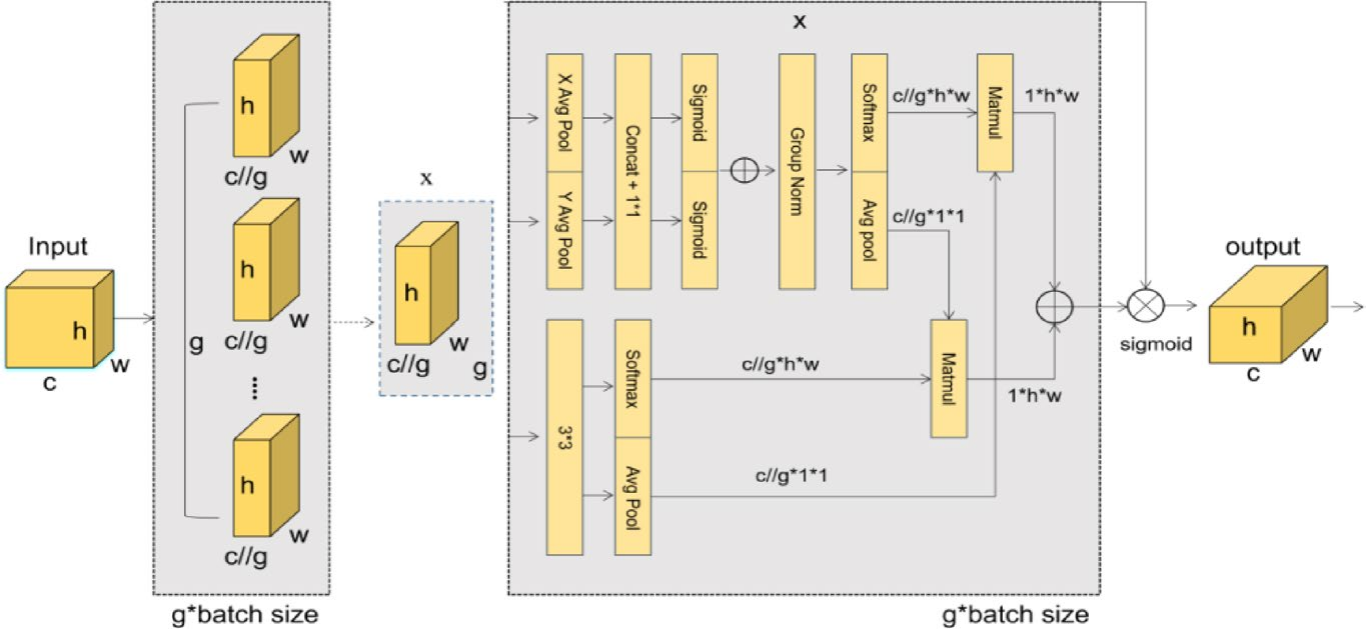
Structure of EMA attention module.

In the Efficient Multi-scale Attention mechanism^20^(As show in Fig. 3), the input information X extracts the attention weight descriptors of the grouped feature maps through three parallel routes. In the two 1 × 1 branches, the channels are encoded along the X and Y spatial directions respectively. The average pooling (Avg Pool) operation is adopted to obtain *(x_h)* and *(x_w)*. Through channel interaction operations, *(x_h)* and *(x_w)* are activated by the Sigmoid function, and then multiplied with the original grouped features (group_x). After that, it is processed by Group Normalization (GN), the weights are calculated through adaptive average pooling and the Softmax function, and finally, the features or weights are merged by matrix multiplication. In the 3 × 3 branch, a 3 × 3 kernel is stacked, the G groups are reshaped and arranged into the batch dimension, and the input tensor is re-defined as C//G*H*W. The output features of the two branches are then modulated through the sigmoid function and normalization operations, and finally merged through the cross-dimensional interaction module. The features of the two branches are fused by matrix multiplication to obtain enhanced feature information. The calculation formula for the final output feature map y is shown in formula (4).

4 $$y = \left( {group_{x} \cdot weights. sigmoid()} \right).reshape\left( {b,c,h,w} \right)$$

### Bounding box loss function based on dynamic non-monotonic focusing mechanism

The WIoU v3^21^(Wise-IoU v3) loss function is a loss function for bounding box regression. It incorporates a dynamic non-monotonic mechanism and devises a rational gradient gain allocation strategy. This strategy reduces large or harmful gradients that occur in extreme samples, thereby enhancing the model’s generalization ability and overall performance. The specific formula is as follows:

5 $$L_{WIoU} = 1 - IoU + \alpha \cdot \frac{{d^{2} }}{{c^{2} }} + \beta \cdot \frac{{\rho^{2} }}{{c^{2} }}$$

where, *IoU* is the Intersection over Union between the predicted bounding box and the ground-truth bounding box. *d* is the Euclidean distance between the centers of the predicted bounding box and the ground-truth bounding box. *c* is the diagonal length of the smallest enclosing box that contains both the predicted bounding box and the ground-truth bounding box. *ρ* represents the difference in the aspect ratio between the predicted bounding box and the ground-truth bounding box. *α* and *β* are weight parameters used to balance the losses of different components.

### PEW_YOLOv8

**Fig. 4:**
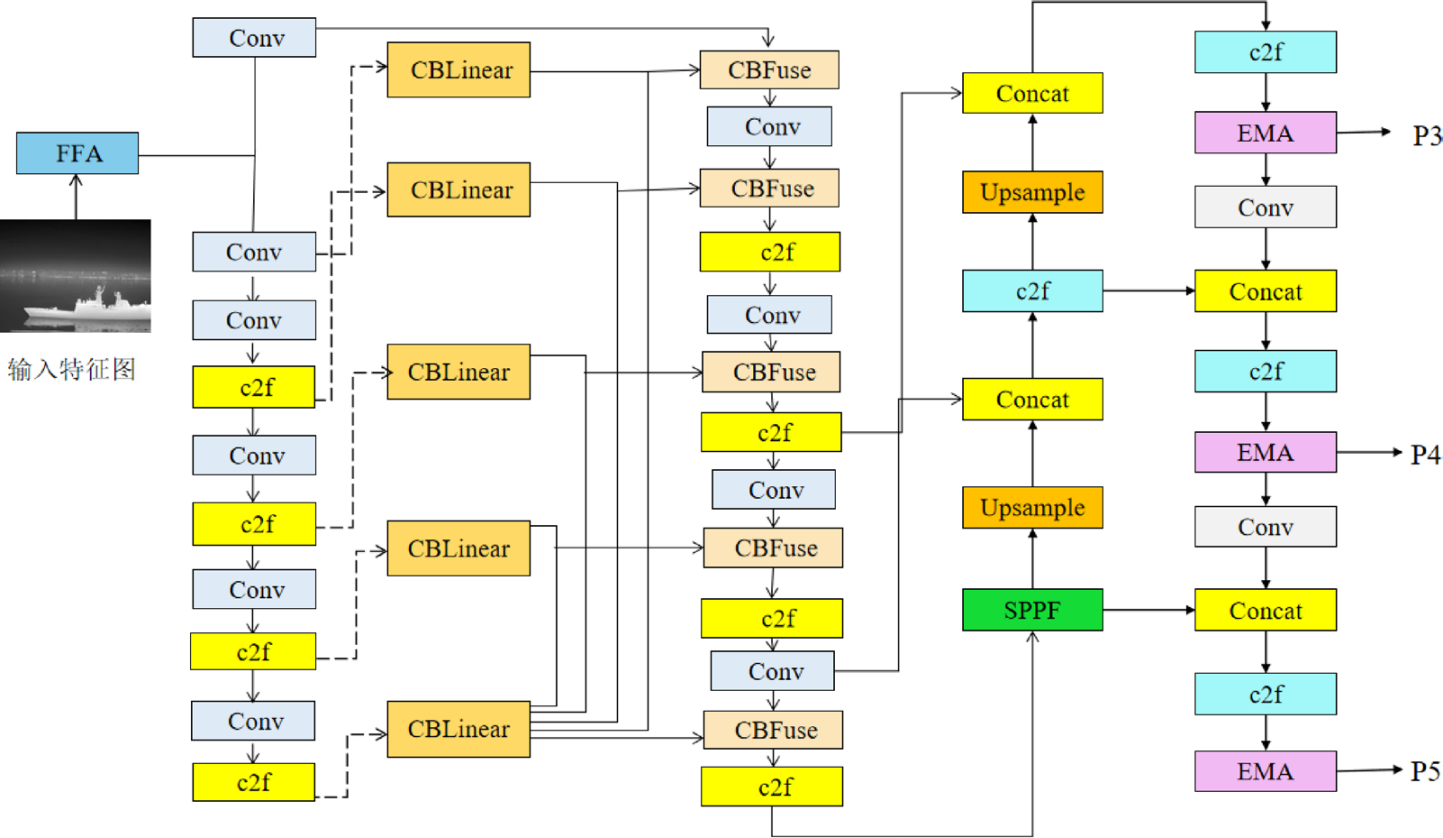
PEW_YOLOv8.

In this study, taking YOLOv8 as the baseline model, aiming at the problems in the infrared ship target detection task, such as poor image quality, low resolution leading to insufficient capture of key features, inadequate multi-scale feature fusion, and interference of target overlap and occlusion on detection accuracy, the PEW_YOLOv8 algorithm is proposed through targeted improvements to the original network structure and functional modules. Its network structure is shown in Fig. 4. The specific improvement strategies are as follows: First, the FFA-Net feature fusion attention network is introduced to repair quality defects such as fog and blurring in the dataset images, thus restoring image details. Second, the PGIG-Backbone feature extraction network is proposed to optimize the gradient transfer efficiency, enabling the network to obtain more reliable gradient information and key features to address the problem of low-resolution infrared ship images in complex scenarios. Then, the Efficient Multi-scale Attention mechanism (EMA Attention) is introduced. By encoding global information, it finely calibrates the channel weights of parallel branches, enhancing the feature representation ability to solve the problem of insufficient multi-scale feature fusion in the neck network. Finally, the Wise IoU v3 bounding box loss function is introduced, which adopts a dynamic non-monotonic focusing mechanism to achieve more fine-grained and effective anchor box quality evaluation and gradient gain allocation.

## Results

### Datasets

The ship target dataset used in this experiment is from the Raytron Technology database. This database contains infrared ship target samples in real-world coastal defense scenarios, which can effectively support the performance verification of target detection algorithms in coastal defense scenarios.

The dataset covers application conditions of multiple scenarios, time periods and resolutions. It includes typical coastal defense scenarios such as open sea waters, port berthing areas, and coastal inshore zones. The target categories include 7 types in total, namely liner, bulk carrier, warship, sailboat, canoe, container ship, and fishing boat. There are 9,400 infrared images in total. The image resolutions are in three specifications: 384 × 288, 640 × 512, and 1280 × 1024.

For the seven types of ship targets in the dataset, in this experiment, rectangular bounding boxes are used to label the targets. Taking the upper-left corner of the image as the coordinate origin [0, 0], the position of the rectangular bounding box is recorded in the form of [× 1, y1, × 2, y2]. Here, × 1 represents the abscissa of the upper-left corner of the rectangular bounding box, y1 represents the ordinate of the upper-left corner of the rectangular bounding box, × 2 represents the abscissa of the lower-right corner of the rectangular bounding box, and y2 represents the ordinate of the lower-right corner of the rectangular bounding box.

To ensure the effectiveness of model training and the objectivity of evaluation, the dataset is randomly divided into a training set, a validation set, and a test set at a ratio of 7:1:2. The training set contains 6,580 images and is used for iterative optimization of model parameters. The validation set contains 940 images and is used to monitor the model performance during training and adjust hyperparameters. The test set contains 1,880 images and is used to independently evaluate the final detection accuracy and generalization ability of the model.

### Experimental setup

This experiment is conducted on the Windows 10 operating system, using a graphics card of model RTX 3080. The software configurations include Anaconda3 2024.06-1 (https://www.anaconda.com/) and PyCharm 2024.2.1 (https://www.jetbrains.com/zh-cn/pycharm/). The Python version is 3.8 (https://www.python.org/downloads/release/python-380/), and the PyTorch version is 1.11.0 (https://pytorch.org/get-started/previous-versions/) +cu113 (https://developer.nvidia.com/cuda-toolkit-archive).

To ensure the reproducibility of the experiment, the core training hyperparameters of this experiment are set as shown in Table 1.

**Table 1:** Hyperparameter setting.

Hyperparameter	Value
Patience	50
Batch	8
Imgsz	640
Pretrained	False
Optimizer	SGD
Ir0	0.01

After comparing the results of using pre-trained weights and not using pre-trained weights, the method of not using pre-trained weights was chosen. The number of training epochs was set to 100. The evaluation metric adopted was the mean average precision at an Intersection over Union (IoU) threshold of 0.5, denoted as mAP50.

6 $${\text{AP 50 }} = \frac{1}{{\mathrm{n}}}\sum\nolimits_{{i = 1}}^{n} {P_{j}^{{IoU = 0.5}} } \left( {R_{i}^{{IoU = 0.5}} } \right)$$

Among them, *P*: represents the precision rate, which is the ratio of the number of correctly predicted positive samples (True Positives, TP) to the number of all samples predicted as positive, representing the accuracy rate. *R* represents the recall rate, which is the ratio of the number of correctly predicted positive samples (True Positives, TP) to the number of all actual positive samples, representing the recall rate. *AP50* refers to the Average Precision (AP) of all classes when the Intersection over Union (IoU) threshold is set to 50%.

### Comparison of different defogging effects

To verify the advantages and disadvantages of the defogging algorithm selected in this experiment compared with other defogging algorithms on the original network, this paper selects three popular image defogging algorithms, namely MB-TaylorFormer, UnfogNet, and AOD-PONO-Net, for comparative experiments. The experimental results are shown in Table 2.

**Table 2:** Comparison of defogging algorithms.

Model	Module	mAP@50(%)	mAP@50:95(%)
YOLOv8	–	88.3	63.3
	MB-TaylorFormer	87.8	62.7
	UnfogNet	88.1	63.2
	AOD-PONO-Net	88.5	63.4
	FFA-Net	**88.7**	**63.9**

Significant values are in bold.

From the above table, it can be seen that not all defogging algorithms are effective. For example, after adding the MB-TaylorFormer module and the UnfogNet module, the mAP of the algorithm decreases. After adding the AOD-PONO-Net module and the FFA-Net module to the algorithm, the accuracy increases, with mAP50 rising by 0.22% and 0.56% respectively. Therefore, the FFA-Net module is selected as the defogging module for this experiment.

### Comparison of different feature extraction networks

To verify the effectiveness of the PGIG-Backbone feature extraction network module proposed in this paper, a comparative experiment was conducted on the baseline network model and the improved model using the dataset. The experimental results are shown in Table 3.

**Table 3:** Comparison of feature extraction networks.

Model	P(%)		R(%)	mAP50(%)	mAP50:95(%)
Baseline	91.4		84.6	88.3	63.3
PGIG-Backbone	**92.9**		**85.8**	**90.8**	**64.9**

Significant values are in bold.

As can be seen from the above table, compared with the baseline model, the mAP50 of the PGIG-Backbone network has increased by 2.5%, and the mAP50:95 has increased by 1.6%. The detection accuracies have reached 90.8% and 64.9% respectively. This demonstrates the effectiveness of the improved network in the object detection task.

### Comparison of different attention mechanisms

To verify the advantages and disadvantages of the EMA attention mechanism in enhancing the performance of the improved network compared with other attention mechanisms, this paper compares the EMA with three popular attention mechanisms, namely CA^22^, CBAM^23^, and ECA^24^. The experimental results are shown in Table 4.

**Table 4:** Comparison of attention mechanisms.

Model	Module	mAP@50(%)
Improved YOLOv8	–	90.8
	CA	90.6(− 0.2)
	CBAM	90.8(−)
	ECA	90.6(− 0.2)
	EMA	**91.2(+ 0.4)**

Significant values are in bold.

As can be seen from the above table, the EMA attention mechanism exhibits a better performance compared to the CA, CBAM, and ECA attention mechanisms, achieving the highest detection accuracy. Compared with the base model, the mAP of the CA and ECA attention mechanisms decreased by 0.2, the detection accuracy of the CBAM attention mechanism remained unchanged, while the mAP of the EMA attention mechanism increased by 0.4.

### Comparison of different loss functions

To verify the impact of applying different loss functions on the model’s detection accuracy, experiments and comparisons were carried out using four improved models with different loss functions: CIoU, Inner IoU, Shape IoU, and WIoU. The experimental results are shown in Table 5.

**Table 5:** Comparison of Loss Functions.

	Loss function	Map50 (%)	Map50:95 (%)
Improved YOLOv8	CIoU	91.2	65.2
	Inner IoU	90.9(− 0.3)	64.8(− 0.4)
	Shape IoU	91.1(− 0.1)	65.2(− )
	WIoUv1	91.2(–)	65.2(− )
	WIoUv2 (*γ* = 0.5)	91.4(+ 0.2)	65.5(+ 0.3)
	WIoUv3 (*α* = 1.9,*γ* = 3)	**91.7(+ 0.5)**	**65.7(+ 0.5)**

Significant values are in bold.

The experimental results show that when the original loss function CIoU is applied to improve YOLOv8, the mAP50 is 91.2% and the mAP50:95 is 65.2. When the Inner IoU is used to replace the loss function, the mAP50 and mAP50:95 decrease by 0.3 and 0.4 percentage points respectively. Shape IoU reduces the detection accuracy by 0.1 percentage point, while WIoUv1 has the same accuracy as the default CIoU. When WIoUv2 and WIoUv3 are used as loss functions, the accuracy is the highest. The mAP50 reaches 91.4 and 91.7 respectively, an increase of 0.2 and 0.5 percentage points compared with CIoU, and the mAP50:90 increases by 0.3 and 0.5 percentage points respectively. In conclusion, WIoUv3 is selected as the loss function for this experiment.

### Comparative experiments with other models

To verify that the model proposed in this paper has better performance compared with other classic models, the model in this paper was compared with other models on the same dataset. The model in this paper was compared with YOLOv5s, YOLOv7, YOLOv8n, YOLOv8, Faster R-CNN, YOLOv11 on the same dataset. The results are shown in Table 6.

**Table 6:** Comparison of different models.

Model	P(%)	R(%)	Map@0.5(%)	Map@0.5–0.9(%)
YOLOv5s	93.2	86.5	**92.3**	66.4
YOLOv7	91.3	78.4	84.8	60.5
YOLOv8n	90.2	75.8	84.1	60.6
YOLOv8	91.4	84.6	88.3	63.3
Faster R-CNN	91.7	80.2	87.3	62.4
YOLOv11	87.0	83.5	88.0	62.9
PEW_YOLOv8	**93.6**	**86.6**	92.2	**66.4**

Significant values are in bold.

From the comparison results, it can be seen that the algorithm PEW_YOLOv8 proposed in this paper has a better detection performance compared with other models. Compared with the base model, the mAP50 of this paper’s algorithm has increased by 3.9 percentage points, reaching a detection accuracy of 92.2%. Compared with YOLOv8n, it has increased by 8.1 percentage points. Compared with YOLOv11, the detection accuracy Map@50 has increased by 4.2. In addition, compared with YOLOv5s, YOLOv5n, YOLOv7, and Faster R-CNN, the accuracy has increased by 0.4, 1.1, 7.9, and 5.4 percentage points respectively. In summary, the algorithm in this experiment has a good detection effect.

### Ablation experiments

To demonstrate the effectiveness of the FFA-Net module, Spatial Depth-conversion Convolution (SPD Conv), Programmable Gradient Information Structure, Efficient Multi-scale Attention Mechanism (EMA Attention), and WIoU loss function proposed in this paper, we conducted ablation studies to evaluate the impact of each component on the performance of the detection algorithm under the same experimental conditions. These ablation studies used the experimental results of the original YOLOv8 network as the baseline.

As can be seen from Table 7, after adding the FFA-Net module to the base model, mAP50 and mAP50-95 increased by 0.4 and 0.6 percentage points respectively, while the FPS decreased to 105.5. After introducing the PGIG-Backbone feature extraction network, mAP50 and mAP50-95 increased by 2.1 and 1.0 percentage points respectively, and the FPS simultaneously dropped to 90.3. After introducing the efficient multi-scale attention mechanism EMA module, the model accuracies mAP50 and mAP50-95 increased by 0.4 and 0.3 percentage points respectively, with the FPS being 85.6. Finally, to enable the model to locate targets more accurately, by introducing the WIoU loss function, the model accuracies mAP50 and mAP50-95 increased by 1.0 and 1.2% points respectively, and the FPS slightly decreased to 85.2. The simultaneous use of the four improvement strategies increased the model’s mAP to 92.2%, a 3.9—percentage—point increase compared to before the improvement, and mAP50:95 reached 66.4%, a 3.1—percentage—point increase compared to before the improvement. The moderate decrease in FPS did not affect its actual inference efficiency. The moderate decrease in FPS did not affect its actual inference efficiency..

**Table 7:** Ablation experiments.

Model	FFA-Net	PGIG- backbone	EMA	WIoU	Map50 (%)	Map50-95 (%)	FPS
YOLOv8					88.3	63.3	**119.0**
	√				88.7	63.9	105.5
	√	√			90.8	64.9	90.3
	√	√	√		91.2	65.2	85.6
	√	√	√	√	**92.2**	**66.4**	85.2

Significant values are in bold.

### Visualization of results

**Fig. 5:**
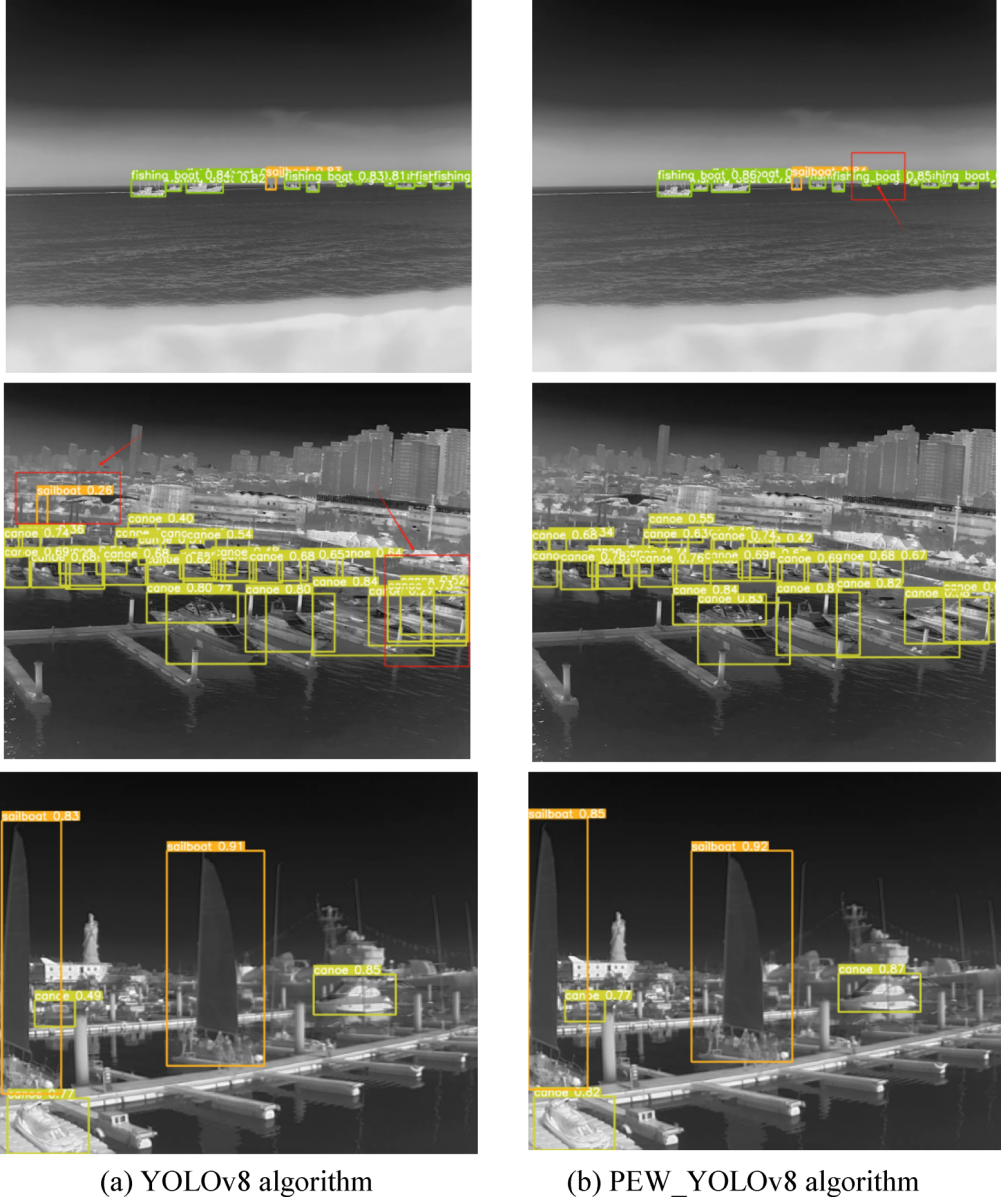
Visualized results.

As can be seen from Fig. 5, in the images of the first row, the base model has insufficient detection ability for small targets and fails to detect the fishing boat in the middle of the image, while our PEW_YOLOv8 algorithm can detect this target. In the images of the second row, false detections occur in the upper-left and lower-right corners of the base model, while the improved algorithm can accurately predict the positions of the ships. In the images of the third row, the detection accuracies of the base model for canoe and sailboat are 0.77, 0.83, 0.49, 0.91, and 0.85 from left to right, and the detection accuracies of the improved algorithm for these targets reach 0.82, 0.85, 0.77, 0.92, and 0.87. Judging from the results, the effectiveness of the PEW_YOLOv8 algorithm is verified.

## Conclusion and future work

### Conclusion

Aiming at the deficiencies of YOLOv8 in the infrared ship detection scenario, this paper proposes an infrared ship detection algorithm PEW_YOLOv8 based on improved YOLOv8, which effectively improves the detection ability of ship targets. The specific improvement measures include:

1. The FFA-Net module is introduced. By combining channel and pixel attention with local residual learning, it restores the image detail information and achieves the image defogging effect.
2. Design the PGIG-Backbone feature extraction network based on the programmable gradient information structure, enabling the model to obtain reliable gradient information for updating network weights and improving detection accuracy.
3. Introduce the EMA attention into the neck network to enhance shallow-and deep-layer features, improving the model’s object-detection ability at multiple scales and the speed of model inference.
4. WIoUv3 is used as the loss function to enhance the network’s attention to ordinary-quality anchor frames.

The PEW_YOLOv8 algorithm proposed in this paper was verified on the Raytron Technology database. Through ablation experiments, it is known that the introduction of the FFA-Net network increased the average detection accuracy of the algorithm by 0.4% and mAP50:95 by 0.6 percentage points. The introduction of the PGIG-Backbone backbone network increased the average detection accuracy of the algorithm by 2.1% and mAP50:95 by 1.0 percentage point. The introduction of the efficient multi-scale attention mechanism EMA increased the average detection accuracy of the algorithm by 0.4% and mAP50:95 by 0.3 percentage points. The introduction of WIoU increased the average detection accuracy of the algorithm by 1.0% and mAP50:95 by 1.2 percentage points. After integrating all the modules, the detection accuracy of the PEW_YOLOv8 algorithm reached 92.2%. Compared with YOLOv8, the average detection accuracy increased by 3.9 percentage points and mAP50:95 increased by 3.1 percentage points.

### Future work

Although the PEW_YOLOv8 algorithm proposed in this paper has achieved remarkable progress in the infrared ship detection task, effectively improving the detection accuracy, there is still room for further optimization and expansion:

1. The current algorithm’s recognition effect for overlapping and occluded ship targets has not reached the optimal level. In complex scenarios where multiple targets are densely stacked or partially occluded, the model is prone to feature confusion. In the future, an occlusion-aware feature decoupling mechanism can be explored. For example, dynamic attention masks or instance-level feature separation modules can be introduced to specifically extract effective features in occluded areas. At the same time, contextual association information can be integrated to assist in target contour and category determination, thereby enhancing the detection robustness in occluded scenarios.
2. There is a need to further balance the relationship between detection accuracy and speed. Although the inference efficiency of the current improved model can meet the requirements of basic scenarios, there is still room for optimization for low-latency scenarios such as real-time maritime monitoring. In the future, efforts can be made from two aspects. On one hand, lightweight network design, model pruning, and quantization techniques can be adopted to compress the scale of network parameters. On the other hand, feature reuse and parallel computing strategies can be explored to reduce redundant calculations without losing key features, thus improving the inference speed while ensuring the detection accuracy.

In addition, in the future, this algorithm can also be applied to more practical maritime monitoring scenarios (such as ship detection at night or in harsh sea conditions) to verify its generalization ability, providing more reliable technical support for practical applications such as maritime traffic safety and maritime management.

## Data Availability

The data that support the findings of this study are available from Raytron Technology Co., Ltd. but restrictions apply to the availability of these data, which were used under license for the current study, and so are not publicly available. Data are however available from the authors upon reasonable request and with permission of Raytron Technology Co., Ltd. For data access inquiries, please contact Menglin Zhu at zhumenglin@stu.ncwu.edu.cn.
